# Racial, ethnic, and sex disparities in the utilization and outcomes of tricuspid valve surgery

**DOI:** 10.1097/MS9.0000000000002203

**Published:** 2024-06-19

**Authors:** Mahmoud Ismayl, Hasaan Ahmed, Andrew M. Goldsweig, Mohamad Alkhouli, Mayra Guerrero

**Affiliations:** a Department of Cardiovascular Medicine, Mayo Clinic, Rochester, MN; b Department of Internal Medicine, Creighton University, Omaha, NE; c Department of Cardiovascular Medicine, Baystate Medical Center, Springfield, MA, USA

**Keywords:** racial disparities, sex disparities, tricuspid valve disease, tricuspid valve repair, tricuspid valve replacement

## Abstract

**Background:**

Data on racial/ethnic and sex disparities in the utilization and outcomes of tricuspid valve surgery (TVS) in the United States are scarce. The authors aimed to evaluate the impact of race/ethnicity and sex on the utilization and outcomes of TVS.

**Methods:**

The authors analyzed the National Inpatient Sample database from 2016 to 2020 to identify hospitalizations for TVS. Racial/ethnic and sex disparities in TVS outcomes were determined using logistic regression models.

**Results:**

Between 2016 and 2020, 19 395 hospitalizations for TVS were identified. The utilization rate (number of surgeries/100,000 hospitalizations) was lower in Black and Hispanic patients compared with White patients for surgical tricuspid valve repair (STVr) (331 versus 493 versus 634, *P*<0.01) and surgical tricuspid valve replacement (STVR) (312 versus 601 versus 728, *P*<0.01). Similarly, the utilization rate was lower for women compared with men for STVr (1021 versus 1364, *P*<0.01) and STVR (930 versus 1,316, *P*<0.01). Compared to White men undergoing TVS, all women had lower odds of acute kidney injury [adjusted odds ratio (aOR) 0.65, 95% CI 0.55–0.78] and higher odds of blood transfusion (aOR 1.30, 95% CI 1.07–1.59), and Black men had higher odds of blood transfusion (aOR 1.59, 95% CI 1.08–2.35). In-hospital mortality and other surgical complications were similar between all groups (all *P*>0.05).

**Conclusions:**

Significant racial/ethnic and sex disparities exist in the utilization of TVS in the United States. Further studies are needed to understand the reasons for these disparities and to identify effective strategies for their mitigation.

## Introduction

Highlights

- Tricuspid valve surgery is lower in women, Black, and Hispanic patients compared to White men.
- Women have lower odds of acute kidney injury and higher odds of blood transfusion.
- Black men have higher odds of blood transfusion.
- Black and Hispanic patients have longer LOS and higher hospitalization costs.

Tricuspid valve disease is a prevalent cardiac condition, with tricuspid regurgitation associated with increased mortality risk^1,2^. Tricuspid regurgitation affects nearly two million patients in the US, with a significant portion experiencing coexisting cardiovascular morbidities including aortic stenosis and mitral regurgitation^3^. Furthermore, elderly patients with tricuspid regurgitation suffer from recurrent hospitalizations, prolonged lengths of hospital stay, and increased mortality^4^. Given the morbidity and mortality associated with tricuspid valve disease, tricuspid valve surgery (TVS), including both repair and replacement, have increased in frequency in order to restore physiological valve function and enhance survival^1^.

The US continues to face challenges of racial/ethnic and sex disparities in healthcare, with the American Heart Association highlighting root factors of both institutional racism and socioeconomic inequality^5,6^. While there have been significant advancements in the effectiveness of cardiac procedures, underrepresented minorities continue to face obstacles in obtaining these interventions while remaining susceptible to adverse outcomes after procedural interventions^7^. Despite the increasing prevalence of TVS, the impact of racial/ethnic and sex disparities on both surgical tricuspid valve repair (STVr) and replacement (STVR) have not yet been examined.

## Material and methods

### Data source and ethics statement

Hospitalization data were abstracted from the National Inpatient Sample (NIS) database, which is part of the Healthcare Cost and Utilization Project (HCUP) family of databases sponsored by the Agency for Healthcare Research and Quality^8^. The NIS is the largest publicly available, fully deidentified, all‐payer inpatient healthcare database in the US. The NIS is derived from billing data submitted by hospitals to statewide organizations across the US and has reliable and verified patient linkage numbers that can be used to track patients across hospitals within each state while adhering to strict privacy guidelines. The NIS database contains both patient‐ and hospital‐level information from ~1000 hospitals and represents ~20% of all US hospitalizations, covering >7 million unweighted hospitalizations each year. When weighted, the NIS extrapolates to the national level, representing 35 million hospitalizations each year. The NIS is compiled annually, which allows for the analysis of procedure trends over time^9^. This study was exempt from the requirements of the Mayo Clinic Institutional Review Board (IRB) because the NIS is a publicly available database comprised of deidentified data. This study has been reported in line with the STROCSS criteria^10^. Supplemental Digital Content 1, http://links.lww.com/MS9/A510.

### Study population and patient selection

**Figure 1:**
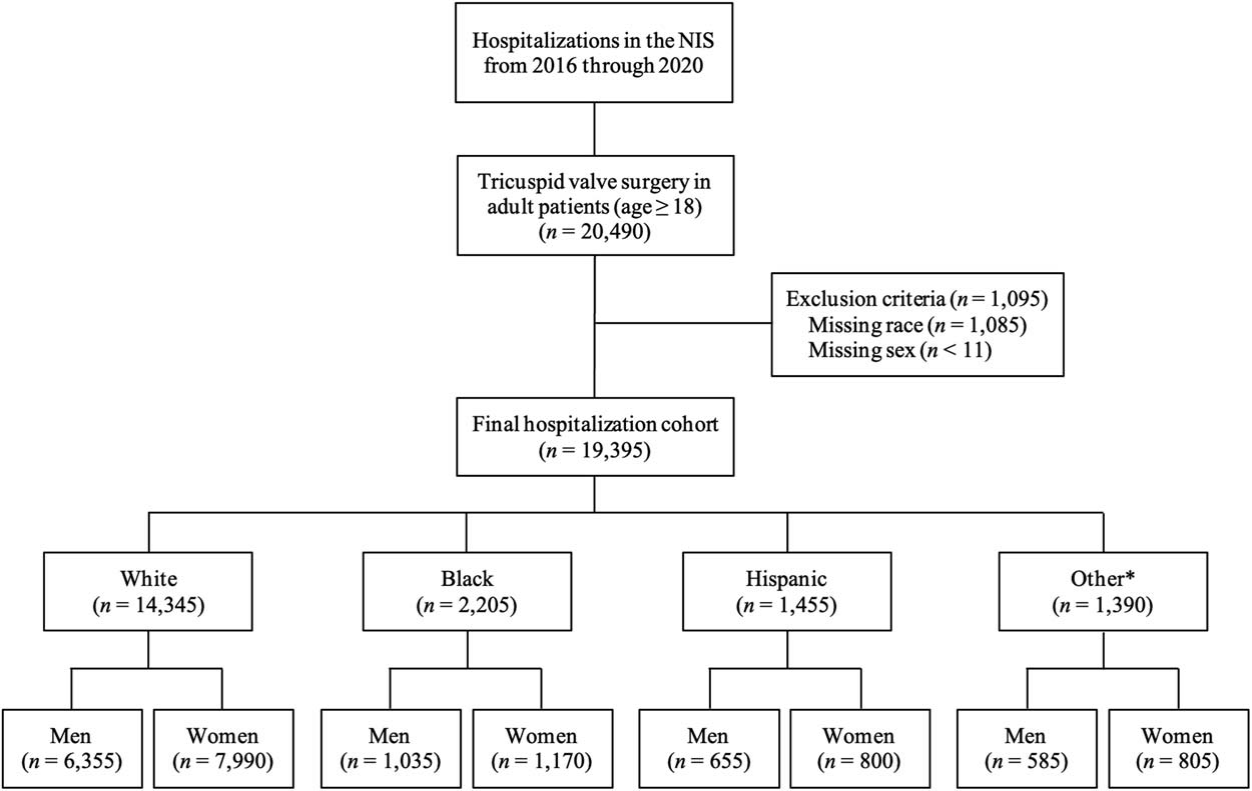
Study flow diagram. Hospitalization counts represent national-level estimates. *Asian or Pacific Islander, Native American, and Other. NIS, National Inpatient Sample.

We queried the NIS database from January 2016 through December 2020 to identify hospitalizations in which adult patients underwent STVr or STVR. We excluded hospitalizations in which the patient was aged younger than 18 years as well as those with missing data on race/ethnicity or sex. For hospitalizations that met inclusion criteria, we stratified the total cohort by race/ethnicity (White, Black, Hispanic, and other) as well as sex (Fig. 1). NIS combines “race” and “ethnicity” into 1 data element (race). If both “race” and “ethnicity” were available, HCUP preferred ethnicity over race in assigning a value for the “race” variable^11^. Similar to prior NIS studies, 3 racial/ethnic groups with small sample sizes (Asian or Pacific Islander, Native American, and Other) were combined into a single “other” group to facilitate the analysis^12,13^. The other 3 HCUP race/ethnicity groups (White, Black, and Hispanic) were left unchanged for the study. All *ICD-10* diagnosis and procedure codes used in this study are presented in Table S1, Supplemental Digital Content 2, http://links.lww.com/MS9/A511.

### Study outcomes

The primary outcome was TVS utilization rate per race/ethnicity and sex defined as the number of TVS performed per 100 000 US hospitalizations. Secondary outcomes included in‐hospital mortality, heart block, permanent pacemaker (PPM) insertion, stroke, acute kidney injury (AKI), major bleeding, need for blood transfusion, and vascular complications (defined as a composite of arteriovenous fistula, aneurysm, hematoma, retroperitoneal bleeding, and venous thromboembolism). We also evaluated hospital length of stay (LOS), total costs (inflation adjusted to 2020 US dollars)^14^, and discharge disposition. Charge-to-cost ratio files were used to convert charges to costs at the individual hospital level. The temporal trends in STVr and STVR use were also assessed. A subgroup analysis was performed to compare the outcomes of isolated TVS in different racial/ethnic and sex groups by excluding concomitant mitral, aortic, and pulmonic valve surgeries and coronary artery bypass grafting.

### Statistical analysis

Categorical variables were compared using the Pearson χ^2^ test. Continuous variables were compared using the Kruskal–Wallis one‐way ANOVA. We reported categorical variables as percentages and continuous variables as medians with interquartile ranges.

A multivariable logistic regression analysis was constructed to adjust for potential confounders, which included age, insurance, income, hospital location and teaching status, bed size, region, type of admission (elective/non-elective and weekend/weekday), Elixhauser and Charlson comorbidity index scores, relevant comorbidities, and concomitant surgeries (Table S2, Supplemental Digital Content 3, http://links.lww.com/MS9/A512). Adjustment variables were selected a priori on the basis of their clinical significance, which may directly influence in‐hospital outcomes. The results from these models are presented as adjusted odds ratios (aORs) with 95% CIs. Trend analyses from 2016 through 2020 were conducted using linear regression.

In accordance with the HCUP data use agreement, we did not report variables that contained a small number of observed (i.e. unweighted) hospitalizations (<11) as this could pose a risk of patient identification or data privacy violation^15^. The inability to report is denoted by “NR.” A two‐tailed *P* less than 0.05 was considered statistically significant. All statistical analyses were performed using Stata version 17 (StataCorp) software, accounting for the NIS sampling design, and were weighted using sampling weights provided with the NIS database to estimate national‐level effects per HCUP‐NIS recommendations^9^.

## Results

### Utilization rate of TVS per race/ethnicity and sex

**Figure 2:**
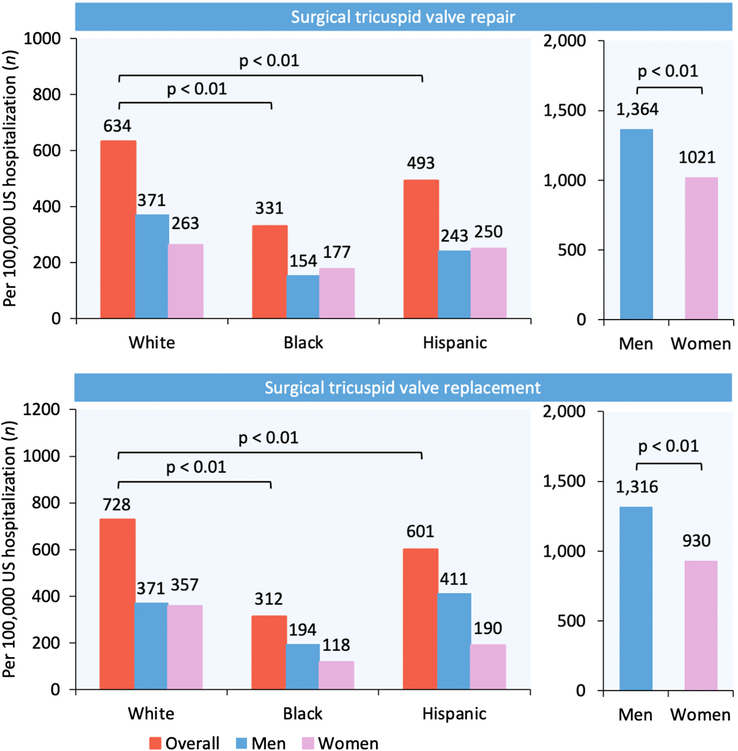
Racial/ethnic and sex differences in the utilization of surgical tricuspid valve repair and replacement in the US.

Among 19 395 weighted hospitalizations for TVS identified in our analysis, 14 345 (73.9%) patients were of White race, 2205 (11.4%) were of Black race, 1455 (7.5%) were of Hispanic ethnicity, 1390 (7.2%) were of other races, and 10 765 (55.5%) were women (Fig. 1). The utilization rate (number of surgeries/100 000 US hospitalizations) was significantly lower in Black and Hispanic patients compared with White patients for STVr (331 versus 493 versus 634, *P*<0.01) and STVR (312 versus 601 versus 728, *P*<0.01; Fig. 2). Similarly, the utilization rate was lower for women compared with men for STVr (1021 versus 1364, *P*<0.01) and STVR (930 versus 1316, *P*<0.01; Fig. 2). After adjustment for potential confounders using multivariable regression analysis, Black race (aOR 0.54, 95% CI 0.47–0.62) and Hispanic ethnicity (aOR 0.71, 95% CI 0.61–0.82) were independently associated with lower TVS utilization compared with White race.

### Baseline risk profile among racial/ethnic and sex groups

There were notable differences in demographic and clinical characteristics across the racial/ethnic and sex groups. Black patients undergoing TVS were younger compared with patients of other racial/ethnic groups. Black patients and Hispanic women had higher Elixhauser and Charlson comorbidity index scores compared to White patients. Differences were also found in the socioeconomic status of each racial/ethnic and sex group, with more women, Black, and Hispanic patients living in neighborhoods in the lowest quartile of median household income compared to White men. Baseline characteristics stratified by race/ethnicity and sex are shown in Tables 1 and 2.

**Table 1:** Demographic and hospital characteristics stratified by race/ethnicity and sex

	White		Black		Hispanic		Other*		
	Men (*n*=6355)	Women (*n*=7990)	Men (*n*=1035)	Women (*n*=1170)	Men (*n*=655)	Women (*n*=800)	Men (*n*=585)	Women (*n*=805)	*P*
Demographic characteristics									
Age (years)	59 (38–72)	51 (31–72)	48 (35–62)	50 (35–64)	57 (44–62)	59 (40–68)	60 (45–69)	62 (42–71)	<0.01
18–64	59.3	63.2	79.4	75.6	79.2	66.3	64.1	59.6	<0.01
65–74	22.0	17.3	11.5	17.9	16.9	20.0	24.8	24.2	
75–84	17.5	17.6	5.3	6.0	3.9	13.7	9.4	15.5	
85+	1.2	1.8	3.8	NR^a^	NR^a^	NR^a^	NR^a^	NR^a^	
Insurance									
Medicare	44.9	40.9	41.9	46.3	23.2	40.5	42.0	45.9	<0.01
Medicaid	23.5	32.4	27.7	31.7	40.0	35.3	21.4	25.5	
Private insurance	27.0	20.9	28.3	19.8	29.6	22.2	32.1	24.8	
Self-pay	4.6	5.7	2.1	2.2	7.2	2.0	4.5	3.8	
Income quartile^b^									
I	26.5	29.8	54.4	51.3	40.5	40.6	18.9	20.9	<0.01
II	25.8	27.7	19.1	21.3	18.2	27.1	24.3	24.1	
III	25.0	23.7	13.7	16.5	27.3	19.4	24.3	24.7	
IV	22.7	18.8	12.7	10.9	14.0	12.9	32.4	30.4	
Hospital characteristics									
Location/teaching status									
Rural	2.4	2.1	NR^a^	NR^a^	NR^a^	NR^a^	NR^a^	NR^a^	0.07
Urban nonteaching	6.5	7.4	3.4	8.5	5.3	4.4	3.4	8.7	
Urban teaching	91.0	90.6	96.1	90.6	94.7	95.6	96.6	91.3	
Bed size^c^									
Small	8.3	7.8	4.3	6.4	5.3	6.9	8.5	8.1	0.03
Medium	20.4	21.5	17.4	12.8	13.0	15.6	16.2	14.9	
Large	71.3	70.7	78.3	80.8	81.7	77.5	75.2	77.0	
Region									
Northeast	17.0	16.2	17.9	17.5	20.6	19.4	34.2	30.4	<0.01
Midwest	30.3	30.5	28.0	18.4	9.2	8.7	12.0	8.1	
South	34.1	38.3	46.9	52.1	26.0	31.2	24.8	22.4	
West	18.6	15.0	7.2	12.0	44.3	40.6	29.1	39.1	
Elective admission	50.1	47.5	33.3	44.9	37.2	60.6	45.3	57.8	<0.01
Weekend admission	12.7	12.5	20.3	12.0	16.0	10.0	13.7	10.6	0.06

*Asian or Pacific Islander, Native American, and Other.

Data presented as median (IQR) or %.

IQR, interquartile range.

a Cell counts <11 are not reportable (NR) per HCUP guidelines.

b Estimated median household incomes are ZIP code–specific, updated annually, and classified into 4 quartiles indicating the poorest to wealthiest populations.

c Bed-size categories are based on inpatient beds and are specific to the hospital’s location and teaching status. A more detailed explanation of all the variables in the NIS, including the specific dollar amounts in each category of median household income and the number of hospital beds in each category, is available online (https://www.hcup-us.ahrq.gov/db/nation/nis/nisdde.jsp).

**Table 2:** Clinical characteristics stratified by race/ethnicity and sex

	White		Black		Hispanic		Other*		
	Men (*n*=6355)	Women (*n*=7990)	Men (*n*=1035)	Women (*n*=1170)	Men (*n*=655)	Women (*n*=800)	Men (*n*=585)	Women (*n*=805)	*P*
Elixhauser comorbidity index	6 (5–8)	6 (5–8)	7 (6–8)	7 (5–8)	6 (4–7)	7 (5–8)	6 (5–8)	6 (5–8)	<0.01
Charlson comorbidity index	2 (1–3)	1 (1–3)	3 (1–5)	2 (1–4)	1 (0–3)	2 (1–4)	2 (1–3)	2 (1–3)	<0.01
0	21.7	23.4	10.6	13.2	26.0	15.6	14.5	15.5	<0.01
1	23.8	32.0	19.8	22.2	26.7	27.5	24.8	30.4	
2	17.9	18.2	15.5	15.8	16.8	20.0	13.7	18.6	
≥3	36.6	26.3	54.1	48.7	30.5	36.9	47.0	35.4	
Individual comorbidities									
Diabetes mellitus	18.3	12.9	32.4	30.3	25.2	26.9	23.1	23.6	<0.01
Hypertension	57.2	47.9	73.9	72.6	55.7	62.5	68.4	55.9	<0.01
Dyslipidemia	35.6	25.4	26.6	35.5	29.0	29.4	36.8	33.5	<0.01
Nicotine/tobacco use	44.1	46.0	37.7	30.8	36.6	26.9	35.9	18.0	<0.01
Alcohol abuse	5.6	2.2	4.3	1.3	5.3	NR^a^	5.1	NR^a^	<0.01
Drug abuse	23.7	35.9	16.4	15.8	24.4	16.9	10.3	12.4	<0.01
Obesity	11.2	13.8	12.6	25.6	12.2	14.4	13.7	13.0	<0.01
Coronary artery disease	35.0	20.8	39.6	29.1	23.7	30.0	40.2	32.9	<0.01
Peripheral vascular disease	13.2	9.4	18.8	15.8	3.8	11.9	12.0	13.7	<0.01
Atrial fibrillation/flutter	52.2	42.4	43.5	42.7	38.9	50.0	59.0	60.9	<0.01
Congestive heart failure	59.1	53.1	72.9	70.9	62.6	65.6	70.1	62.7	<0.01
Renal failure	26.0	17.1	43.5	33.3	22.9	23.1	28.2	24.2	<0.01
Dialysis dependent	1.8	1.3	10.1	7.3	6.1	4.4	6.8	2.5	<0.01
Liver disease	15.2	17.0	18.4	9.8	18.3	16.9	17.9	16.1	0.20
Chronic pulmonary disease	19.4	20.2	21.7	23.1	13.0	15.0	17.9	15.5	0.18
Obstructive sleep apnea	14.0	7.3	12.6	11.5	8.4	10.0	7.7	5.0	<0.01
Coagulopathy	45.4	42.6	51.7	40.2	45.8	51.9	42.7	43.5	0.08
Cancer	2.3	1.1	NR^a^	1.3	NR^a^	NR^a^	NR^a^	NR^a^	0.08
Malnutrition	11.9	11.8	14.5	9.8	15.3	11.9	4.3	10.6	0.16
Dementia	0.6	0.5	NR^a^	NR^a^	NR^a^	NR^a^	NR^a^	NR^a^	0.70
Depression	10.0	20.1	10.1	16.2	10.7	11.9	5.1	8.1	<0.01
Previous history									
Myocardial infarction	5.1	2.6	6.8	4.7	3.8	5.0	4.3	3.1	0.01
Stroke/TIA	4.6	4.5	5.3	4.7	4.6	10.0	3.4	6.8	0.11
Cardiac arrest	0.8	0.6	NR^a^	1.3	NR^a^	NR^a^	NR^a^	NR^a^	0.80
PCI	6.0	2.5	4.3	3.0	4.6	5.0	2.6	5.0	<0.01
CABG	4.2	2.5	1.9	NR^a^	5.3	1.9	4.3	3.1	0.02
ICD	3.9	2.1	6.8	6.4	NR^a^	NR^a^	4.3	2.5	<0.01
PPM	5.7	7.8	1.9	3.8	6.9	6.9	6.0	9.3	0.01
Concomitant surgery									
Mitral valve surgery	25.6	26.6	23.2	39.7	25.2	40.6	33.3	42.2	<0.01
Pulmonic valve surgery	3.1	1.8	2.9	NR^a^	4.6	NR^a^	NR^a^	3.7	0.05
Aortic valve surgery	16.8	12.0	15.5	17.5	11.5	10.6	13.7	14.9	0.01
CABG	15.9	7.3	13.5	10.3	11.9	9.9	17.1	14.3	<0.01

*Asian or Pacific Islander, Native American, and Other.

Data presented as median (IQR) or %. Two authors (M.I. and H.A.) independently verified the *International Classification of Diseases, Tenth Revision* (*ICD‐10*) codes that corresponded to each comorbidity (*Table S1*, Supplemental Digital Content 2, http://links.lww.com/MS9/A511), and any disagreements in inclusion or exclusion of *ICD-10* codes were discussed with a third author (A.M.G).

CABG, coronary artery bypass grafting; ICD, implantable cardioverter-defibrillator; IQR, interquartile range; PCI, percutaneous coronary intervention; PPM, permanent pacemaker; TIA, transient ischemic attack.

a Cell counts <11 are not reportable (NR) per HCUP guidelines.

### In-hospital outcomes of TVS stratified by race/ethnicity and sex

After adjustment for potential confounders using multivariable regression analysis, women had lower odds of AKI (aOR 0.65, 95% CI 0.55–0.78, *P*<0.01) and higher odds of blood transfusion (aOR 1.30, 95% CI 1.07–1.59, *P*<0.01) compared to White men. Black men had higher odds of blood transfusion (aOR 1.59, 95% CI 1.08–2.35, *P*<0.01) compared to White men. In-hospital mortality, heart block, need for permanent pacemaker, stroke, major bleeding, and vascular complications were similar between all groups (all *P*>0.05).

Black and Hispanic patients had a longer hospital LOS and higher total costs compared with White patients. For hospitalizations in which the patient was discharged alive, women, Black, and Hispanic patients had lower rates of home discharges as opposed to non-home discharges compared to White men. In‐hospital outcomes stratified by race/ethnicity and sex are shown in Table 3.

**Table 3:** In-hospital outcomes stratified by race/ethnicity and sex

	White		Black		Hispanic		Other*		
	Men (*n*=6355)	Women (*n*=7990)	Men (*n*=1035)	Women (*n*=1170)	Men (*n*=655)	Women (*n*=800)	Men (*n*=585)	Women (*n*=805)	*P*
Death									
%	7.7	5.3	7.2	6.4	6.9	6.9	6.8	14.3	**<0.01**
aOR (95% CI)	Ref.	0.87 (0.61–1.24)	0.78 (0.38–1.56)	1.05 (0.57–1.94)	1.12 (0.49–2.57)	0.91 (0.42–1.98)	0.75 (0.31–1.81)	1.73 (0.96–3.13)	—
Heart block									
%	28.4	31.9	28.0	29.5	25.2	30.6	25.6	28.0	0.38
aOR (95% CI)	Ref.	1.01 (0.84–1.22)	0.94 (0.66–1.33)	0.91 (0.63–1.29)	0.79 (0.48–1.32)	0.96 (0.63–1.46)	0.89 (0.56–1.42)	0.95 (0.65–1.39)	—
Permanent pacemaker									
%	13.6	17.1	13.5	15.0	11.5	15.0	12.8	17.4	0.18
aOR (95% CI)	Ref.	1.19 (0.95–1.49)	1.03 (0.64–1.65)	0.96 (0.61–1.50)	0.85 (0.48–1.49)	0.93 (0.54–1.59)	1.00 (0.53–1.88)	1.26 (0.75–2.13)	—
Stroke									
%	4.8	4.4	6.3	4.7	3.8	6.3	2.6	7.5	0.51
aOR (95% CI)	Ref.	0.95 (0.62–1.47)	0.78 (0.35–1.75)	0.73 (0.30–1.76)	1.11 (0.33–3.70)	1.43 (0.55–3.68)	0.17 (0.02–1.66)	2.05 (0.97–4.36)	—
Acute kidney injury									
%	45.2	37.5	55.6	34.2	44.3	27.5	45.3	34.2	**<0.01**
aOR	Ref.	**0.70 (0.58**–**0.84)**	1.24 (0.84–1.83)	**0.56 (0.38**–**0.82)**	1.04 (0.67–1.60)	**0.43 (0.28**–**0.64)**	1.13 (0.74–1.72)	**0.55 (0.36**–**0.84)**	—
Major bleeding									
%	59.4	57.3	66.7	54.7	56.5	55.6	59.0	61.5	0.23
aOR (95% CI)	Ref.	0.91 (0.77–1.07)	1.28 (0.88–1.86)	0.77 (0.57–1.04)	0.95 (0.62–1.47)	0.82 (0.56–1.20)	0.90 (0.58–1.39)	1.14 (0.78–1.67)	—
Blood transfusion									
%	21.2	24.8	28.5	29.5	26.7	31.3	27.4	32.9	**<0.01**
aOR (95% CI)	Ref.	**1.35 (1.01**–**1.79)**	**1.59 (1.08**–**2.35)**	**1.60 (1.10**–**2.32)**	1.35 (0.84–2.17)	**1.83 (1.21**–**2.77)**	1.62 (0.98–2.62)	**1.92 (1.26**–**2.91)**	—
Vascular complications									
%	5.7	5.3	7.2	6.0	6.1	9.4	5.1	4.3	0.54
aOR (95% CI)	Ref.	0.81 (0.57–1.17)	0.81 (0.41–1.58)	0.87 (0.43–1.75)	1.03 (0.47–2.26)	1.34 (0.68–2.64)	0.74 (0.29–1.87)	0.75 (0.32–1.75)	—
Discharge disposition									
Routine	36.2	31.6	27.1	26.9	30.5	27.5	47.9	28.0	**<0.01**
Transfer to short-term hospital	2.3	3.3	2.4	1.3	3.8	1.9	2.6	1.9	
Transfer to SNF or ICF	25.6	33.8	30.9	26.1	32.1	33.1	12.8	27.3	
Home healthcare	26.6	23.1	31.9	38.0	24.4	30.0	29.1	28.6	
Resource utilization									
LOS (days)	13 (8–26)	14 (8–29)	21 (13–36)	16 (9–26)	16 (10–30)	15 (9–25)	14 (8–28)	12 (8–20)	**<0.01**
Hospital cost ($)	92 314 (54 034–178 869)	93 194 (56 114–163 942)	132 941 (71 761–274 186)	99 672 (59 934–180 271)	110 661 (72 661–248 005)	118 044 (60 431–224 442)	104 033 (59 607–226 389)	101 050 (61 995–179 517)	**<0.01**

*Asian or Pacific Islander, Native American, and Other.

Bold values indicate statistical significance.

Data presented as median (IQR), %, or aOR (95% CI). The *International Classification of Diseases, Tenth Revision* (*ICD‐10*) codes corresponding to each of the in-hospital outcomes were identified with the same process used to identify comorbidity codes (*Table S1*, Supplemental Digital Content 2, http://links.lww.com/MS9/A511).

aOR, adjusted odds ratio; CI, confidence interval; ICF, intermediate care facility; IQR, interquartile range; LOS, length of stay; SNF, skilled nursing facility.

### Subgroup analysis

In a subgroup analysis comparing the in-hospital outcomes of isolated TVS in different racial/ethnic and sex groups, women had lower odds of AKI (aOR 0.61, 95% CI 0.48–0.78, *P*<0.01) and Black men had higher odds of blood transfusion (aOR 1.91, 95% CI 1.09–3.34, *P*<0.01) compared to White men. In-hospital mortality, heart block, need for permanent pacemaker, stroke, major bleeding, and vascular complications were similar between all groups (all *P*>0.05). Baseline characteristics and adjusted in-hospital outcomes of isolated TVS stratified by race/ethnicity and sex are shown in Tables S3 and S4, Supplemental Digital Content 4, http://links.lww.com/MS9/A513, Supplemental Digital Content 5, http://links.lww.com/MS9/A514 respectively.

### Temporal trends in TVS use

**Figure 3:**
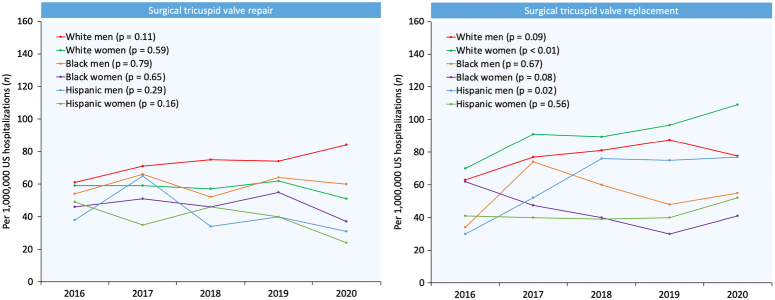
Year-over-year trend in the number of surgical tricuspid valve repair and replacement performed per 1 000 000 hospitalizations in the US in different racial/ethnic and sex groups from 2016 through 2020.

From 2016 through 2020, the number of TVS performed per 1 000 000 hospitalizations remained similar in most racial/ethnic and sex groups, with a significant increase in STVR noted in White women (p_trend_<0.01) and Hispanic men (p_trend_=0.02). Annual trends for STVr and STVR in different racial/ethnic and sex groups are shown in Fig. 3.

## Discussion

We report racial/ethnic and sex disparities among patients undergoing TVS in the US. Our main findings include: (1) the utilization rate of TVS was significantly lower in women, Black, and Hispanic patients compared to White men; (2) compared with White men undergoing TVS, women had lower odds of AKI and higher odds of blood transfusion, and Black men had higher odds of blood transfusion; and (3) Black and Hispanic patients had a longer LOS and higher cost of care compared with White patients.

### Disparities in TVS use

Our study noted a significant underrepresentation of women, Black, and Hispanic patients undergoing TVS. This underrepresentation aligns with previous studies that found that women, Black, and Hispanic patients were significantly underrepresented in cardiac interventions, attributed to inappropriate cultural communication, provider biases impairing both referral and treatment, inadequate healthcare access, and socioeconomic disparities^16–19^. Lower TVS utilization among Black and Hispanic patients compared to White patients highlights the significant healthcare disparities that underrepresented minority groups increasingly face in cardiac surgery, propagated by systematic and patient-level barriers^20^. Despite an increasing prevalence of cardiovascular disease among underrepresented racial/ethnic groups, there remains a significant inequality in both access and referrals to cardiologists and cardiac surgeons among Black and Hispanic patients, compared to White patients, attributed to inadequate insurance coverage, limited availability of specialists in under-resourced communities, risk aversion policies among institutions, and provider biases^20^. Disparities in TVS utilization among Black and Hispanic patients can be further attributed to hesitancy/refusal to undergo cardiac surgery, driven by provider distrust, cultural beliefs, poor healthcare literacy, and financial concerns^20,21^. A multifaceted approach is necessary to attenuate racial/ethnic and sex disparities in TVS use.

As with mitral valve surgery^22–24^, Black patients undergoing TVS were significantly younger compared to patients of other racial/ethnic groups, which could suggest that Black patients have accelerated tricuspid valve disease requiring intervention at a younger age. This could be due to the higher incidence of chronic kidney disease and dialysis dependence in Black patients, which have been associated with tricuspid regurgitation in previous reports^25–27^. Alternatively, perhaps TVS is less frequently offered to older, sicker Black patients compared with White patients.

### In-hospital mortality and complications

Our study found that women undergoing TVS had lower odds of AKI and higher odds of blood transfusion compared to White men, which aligns with prior studies^28,29^. In a retrospective study evaluating AKI rates in a predominantly female cohort of patients undergoing TVS, male sex was an independent predictor for postoperative AKI (aOR 1.73, 95% CI 1.27–2.36)^28^. In a study evaluating outcomes of surgical aortic valve replacement based on sex, Chaker *et al*.^29^ found an increased frequency of blood transfusions in women compared to men (40.4% versus 33.9%, *P*<0.01).

In contrast to concomitant TVS, the odds of blood transfusion in patients undergoing isolated TVS were similar between women and White men. This is congruent with a prior study evaluating sex-stratified outcomes of isolated TVS showing similar postoperative blood transfusions between men and women (34.4% versus 38.5%, *P*=0.30)^30^. Increased blood transfusions in women compared with White men undergoing concomitant TVS noted in our study likely stem from the complexity of multiple procedures involved, resulting in lengthened procedural time and duration of anesthesia, amplifying the risk of blood loss and adverse events^31^. In a prior study evaluating outcomes of isolated versus concomitant TVS, those undergoing concomitant TVS required significantly more blood transfusions than those undergoing isolated TVS (37.9% versus 32.6%, *P*<0.01)^32^.

Black men had higher odds of blood transfusion compared to White men. This is similar to prior studies evaluating racial disparities in coronary artery bypass graft surgery, with Black patients noted to have higher rates of blood transfusion compared to White patients (65.7% versus 62.8%, *P*<0.01)^33^. Increased blood transfusions in Black versus White men can be attributed to the variations in provider practices to initiate transfusion, perpetuated by clinical biases towards minority patients, in addition to socioeconomic disparities^34,35^.

### Hospital LOS and total costs

Our analysis showed that compared to White patients, Black and Hispanic patients undergoing TVS had a longer hospital LOS and higher total costs. This aligns with previous studies evaluating racial disparities in patients undergoing mitral and aortic valve surgeries^22,23,36^. Increased hospital LOS and costs can be attributed to provider biases and practices, as individuals of minority groups have a decreased likelihood of receiving prompt evidence-based care compared to White patients^22,23^. In addition, higher blood transfusions and non-home discharges in Black and Hispanic patients compared to White patients likely played a role in increased hospital LOS and total costs.

### Limitations

Our study has several important limitations. First, in a retrospective NIS study using administrative claims codes, incorrect coding could lead to inaccurate data. Second, the retrospective nature of the study makes it subject to inherent selection bias. Third, some baseline characteristics were not available per the HCUP data use agreement because patient counts were less than 11. Fourth, validated risk scores such as the Society of Thoracic Surgeons (STS) score are not captured by the NIS, limiting patient risk assessment. Fifth, detailed baseline and procedural characteristics such as echocardiographic data, the severity of tricuspid valve disease, surgical incision site, and device type, as well as periprocedural medications were unavailable, which can lead to unmeasured bias. Sixth, the incidence of tricuspid valve disease in different racial/ethnic groups is unknown, and therefore the lower rates of TVS use in Black and Hispanic patients compared to White patients could possibly be related to lower incidence of tricuspid valve disease in these minority groups. Seventh, the NIS allows detailed assessment of in-hospital outcomes but does not include long-term clinical outcomes beyond discharge. Studies exploring the long-term racial/ethnic and sex disparities in outcomes of TVS are still needed.

## Conclusions

Significant racial/ethnic and sex disparities exist in the utilization of TVS in the US. Further studies are needed to understand the reasons for these disparities and to identify effective strategies for their mitigation.

## Ethical approval

This study was exempt from the requirements of the Mayo Clinic Institutional Review Board (IRB) because the NIS is a publicly available database comprised of deidentified data.

## Consent

Consent was not required for this study.

## Source of funding

This research did not receive any specific grant from funding agencies in the public, commercial, or not for-profit sectors.

## Author contribution

Conceptualization: M.I., H.A., A.G., M.A., M.G.; writing—original draft: M.I., H.A., A.G., M.A., M.G.; writing—review and editing: M.I., H.A., A.G., M.A., M.G.; investigation: M.I., H.A., A.G., M.A., M.G.; supervision: A.G., M.A., M.G.

## Conflicts of interest disclosure

The authors have no conflicts of interest to declare.

## Research registration unique identifying number (UIN)

1. Name of the registry: Research registry.

2.Unique identifying number or registration ID: researchregistry10129.

3. Hyperlink to your specific registration: https://www.researchregistry.com/browse-the-registry#home/registrationdetails/6600e64073239b0028f66754/.

## Guarantor

Mahmoud Ismayl, MBBS. E-mail: ismayl.mahmoud@mayo.edu.

## Data availability statement

The NIS data are publicly available for purchase online from the Agency for Healthcare Research and Quality.

## Provenance and peer review

Not commissioned, externally peer-reviewed.

## Supplementary Material
