# Supplementary material for: Racial, ethnic, and sex disparities in the utilization and outcomes of tricuspid valve surgery
Source: Ann Med Surg (Lond). 2024 Jun 19;86(8):4368–76. doi: 10.1097/MS9.0000000000002203 (PMC11305732; doi:10.1097/MS9.0000000000002203)
Supplement: Supplementary file 2 [file ms9-86-4368-s002.docx]

**Table S1.** ICD-10 diagnosis (CM) and procedure (PCS) codes

| **Procedure** | **ICD-10 PCS codes** |
| --- | --- |
| Tricuspid valve surgery | Surgical Tricuspid Valve Repair (STVr):  02QJ0ZG, 02QJ0ZZ  Surgical Tricuspid Valve Replacement (STVR):  02RJ07Z, 02RJ08Z, 02RJ0JZ, 02RJ0KZ |
| Mitral valve surgery | 02RG07Z, 02RG08Z, 02RG0JZ, 02RG0KZ, 02QG0ZE, 02QG0ZZ |
| Pulmonic valve surgery | 02RH07Z, 02RH08Z, 02RH0JZ, 02RH0KZ, 02QH0ZZ |
| Aortic valve surgery | 02RF07Z, 02RF08Z, 02RF0JZ, 02RF0KZ, X2RF032, 02QF0ZJ, 02QF0ZZ |
| CABG | 0210093, 0210098, 0210099, 021009C, 021009F, 021009W, 02100A3, 02100A8, 02100A9, 02100AC, 02100AF, 02100AW, 02100J3, 02100J8, 02100J9, 02100JC, 02100JF, 02100JW, 02100K3, 02100K8, 02100K9, 02100KC, 02100KF, 02100KW, 02100Z3, 02100Z8, 02100Z9, 02100ZC, 02100ZF, 0211093, 0211098, 0211099, 021109C, 021109F, 021109W, 02110A3, 02110A8, 02110A9, 02110AC, 02110AF, 02110AW, 02110J3, 02110J8, 02110J9, 02110JC, 02110JF, 02110JW, 02110K3, 02110K8, 02110K9, 02110KC, 02110KF, 02110KW, 02110Z3, 02110Z8, 02110Z9, 02110ZC, 02110ZF, 0212093, 0212098, 0212099, 021209C, 021209F, 021209W, 02120A3, 02120A8, 02120A9, 02120AC, 02120AF, 02120AW, 02120J3, 02120J8, 02120J9, 02120JC, 02120JF, 02120JW, 02120K3, 02120K8, 02120K9, 02120KC, 02120KF, 02120KW, 02120Z3, 02120Z8, 02120Z9, 02120ZC, 02120ZF, 0213093, 0213098, 0213099, 021309C, 021309F, 021309W, 02130A3, 02130A8, 02130A9, 02130AC, 02130AF, 02130AW, 02130J3, 02130J8, 02130J9, 02130JC, 02130JF, 02130JW, 02130K3, 02130K8, 02130K9, 02130KC, 02130KF, 02130KW, 02130Z3, 02130Z8, 02130Z9, 02130ZC, 02130ZF |

| **Baseline characteristics** | **ICD-10 CM codes** |
| --- | --- |
| **Comorbidities** | |
| DM | E10.0, E10.1, E10.9, E11.0, E11.1, E11.9, E12.0, E12.1, E12.9, E13.0, E13.1, E13.9, E14.0, E14.1, E14.9, E10.2-E10.8, E11.2-E11.8, E12.2-E12.8, E13.2-E13.8, E14.2-E14.8 |
| HTN | I10.x, I11.x-I13.x, I15.x |
| Dyslipidemia | E78.x |
| Nicotine/tobacco use | F17.x, Z72.0, Z87.891 |
| Alcohol abuse | F10, E52, G62.1, I42.6, K29.2, K70.0, K70.3, K70.9, T51.x, Z50.2, Z71.4, Z72.1 |
| Drug abuse | F11.x-F16.x, F18.x, F19.x, Z71.5. Z72.2 |
| Endocarditis | I33.x, I38, I01.1 |
| Obesity | E66.x |
| CAD | I25.x |
| Peripheral vascular disease | I70.x, I71.x, I73.1, I73.8, I73.9, I77.1, I79.0, I79.2, K55.1, K55.8, K55.9, Z95.8, Z95.9 |
| AF/A.flutter | I48.x |
| CHF | I09.9, I11.0, I13.0, I13.2, I25.5, I42.0, 142.5-I42.9, I43.x, I50.x, P29.0 |
| Renal failure | I12.0, I13.1, N18.x, N19.x, N25.0, Z49.0-Z49.2, Z94.0, Z199.2 |
| Dialysis dependent | Z99.2 |
| Liver disease | B18.x, I85.x, I86.4, I98.2, K70.x, K71.1, K71.3-K71.5, K71.7, K72.x-K74.x, K76.0, K76.2-K76.9. Z94.4 |
| Chronic pulmonary disease | I27.8, 127.9, J40.x-J47.x, J60.x-J67.x, J68.4, J70.1, J70.3 |
| OSA | G47.33 |
| Coagulopathy | D65-D68.x, D69.1, D69.3-D69.6 |
| Cancer | C0x.x, C1x.x, C2x.x, C30.x, C31.x, C32.x, C33.x, C34.x, C37.x, C38.x, C39.x, C40.x, C41.x, C43.x, C45.x, C46.x, C47.x, C48.x, C49.x, C50, C51-58.x, C60-63.x, C76.x, C80.1, C81.x, C82.x, C83.x, C84.x, C85.x, C88.x, C9x.x |
| Malnutrition | E43, E44.x, E45, E46 |
| Dementia | F01.x, F02.x, F03.x, F04, F05, F06.1, F06.8, G13.2, G13.8, G30.x, G31.0x, G31.1, G31.2, G91.4, G94, R41.81, R54 |
| Depression | F20.4, F31.3-F31.5, F32.x, F33.x, F34.1, F41.2, F43.2 |
| **Previous history** | |
| MI | I25.2 |
| Stroke/TIA | Z86.73 |
| Cardiac arrest | Z86.74 |
| PCI | Z98.61, Z95.5 |
| CABG | Z95.1 |
| ICD | Z95.810 |
| PPM | Z95.0 |

| **In-hospital outcomes** | **ICD-10 CM/PCS codes** |
| --- | --- |
| **Cardiac** | |
| Heart block | I44.-, I45.0, I45.1-, I45.2, I45.3, I45.4, I45.5 |
| PPM insertion | 0JH634Z, 0JH635Z, 0JH636Z, 0JH63PZ, 0JH834Z, 0JH835Z, 0JH836Z, 0JH83PZ, 0JH604Z, 0JH605Z, 0JH606Z, 0JH60PZ, 0JH804Z, 0JH805Z, 0JH806Z, 0JH80PZ |
| **Neurologic** |  |
| Stroke | I60.-, I61.-, I62.-, I63.-, I67.81, I67.82, G45.-, G46.-, H34.0-, H34.1-, H34.2-, I97.820, I97.810 |
| **Renal** | |
| AKI | N17.-, N99.0 |
| **Hematologic** | |
| Major bleeding | I97610, I97410, I97618, I97418, L7622, L7602, I9751, L7612, K92.0, K92.1, K92.2, K91.841, K91.62, R31.0, N99821, N9962, R04.-, J95831, J9562, R58, D62 |
| Blood transfusion | [30233H0](https://www.icd10data.com/ICD10PCS/Codes/3/0/2/3/30233H0), [30233H1](https://www.icd10data.com/ICD10PCS/Codes/3/0/2/3/30233H1), 30233N0, 30233N1, 30233P0, 30233P1, 30230H0, 30230H1, 30230N0, 30230N1, 30230P0, 30230P1, 30243H0, 30243H1, 30243N0, 30243N1, 30243P0, 30243P1, 30240H0, 30240H1, 30240N0, 30240N1, 30240P0, 30240P1 |
| **Vascular** | |
| Vascular complications | I77.0, I72.-, L7632, L7602, I97630, I97410, I97638, I97418, K66.1, I26.92, I26.93, I26.94, I26.99, I26.02, I26.09, [I82.4](https://www.icd10data.com/ICD10CM/Codes/I00-I99/I80-I89/I82-/I82.4)-, [T81.718A](https://www.icd10data.com/ICD10CM/Codes/S00-T88/T80-T88/T81-/T81.718A), [T81.719A](https://www.icd10data.com/ICD10CM/Codes/S00-T88/T80-T88/T81-/T81.719A), [T81.72XA](https://www.icd10data.com/ICD10CM/Codes/S00-T88/T80-T88/T81-/T81.72XA) |
