# Supplementary material for: Racial, ethnic, and sex disparities in the utilization and outcomes of tricuspid valve surgery
Source: Ann Med Surg (Lond). 2024 Jun 19;86(8):4368–76. doi: 10.1097/MS9.0000000000002203 (PMC11305732; doi:10.1097/MS9.0000000000002203)
Supplement: Supplementary file 3 [file ms9-86-4368-s003.docx]

**Table S2.** Variables used in the multivariable regression analysis to compute adjusted odds of in-hospital outcomes

| **Demographic characteristics** |
| --- |
| Age |
| Insurance |
| Income quartile |
| **Hospital characteristics** |
| Location/teaching status |
| Bed size |
| Region |
| Elective admission |
| Weekend admission |
| **Comorbidities** |
| Elixhauser comorbidity index |
| Charlson comorbidity index |
| Diabetes mellitus |
| Hypertension |
| Dyslipidemia |
| Nicotine/tobacco use |
| Alcohol abuse |
| Drug abuse |
| Endocarditis |
| Obesity |
| Coronary artery disease |
| Peripheral vascular disease |
| Atrial fibrillation/atrial flutter |
| Congestive heart failure |
| Renal failure |
| Dialysis dependent |
| Liver disease |
| Chronic pulmonary disease |
| Obstructive sleep apnea |
| Coagulopathy |
| Cancer |
| Malnutrition |
| Dementia |
| Depression |
| **Previous** **history** |
| Myocardial infarction |
| Stroke/TIA |
| Cardiac arrest |
| PCI |
| CABG |
| ICD |
| PPM |
| **Concomitant surgery** |
| Mitral valve surgery |
| Pulmonic valve surgery |
| Aortic valve surgery |
| CABG |
