# Supplementary material for: Racial, ethnic, and sex disparities in the utilization and outcomes of tricuspid valve surgery
Source: Ann Med Surg (Lond). 2024 Jun 19;86(8):4368–76. doi: 10.1097/MS9.0000000000002203 (PMC11305732; doi:10.1097/MS9.0000000000002203)
Supplement: Supplementary file 4 [file ms9-86-4368-s004.docx]

**Table S3.** Baseline characteristics of isolated tricuspid valve surgery stratified by race/ethnicity and sex

|  | White | | Black | | Hispanic | | Other^a^ | | *p* |
| --- | --- | --- | --- | --- | --- | --- | --- | --- | --- |
|  | Men  (*n*=3,210) | Women  (*n*=4,870) | Men  (*n*=570) | Women  (*n*=550) | Men  (*n*=390) | Women  (*n*=375) | Men  (*n*=265) | Women  (*n*=335) |  |
| **Demographic characteristics** |  |  |  |  |  |  |  |  |  |
| Age | 59 (38-72) | 51 (31-72) | 48 (35-62) | 50 (35-64) | 57 (44-62) | 59 (40-68) | 60 (45-69) | 62 (42-71) | <0.01 |
| 18-64 | 75.9 | 77.3 | 82.1 | 80.0 | 84.2 | 84.0 | 67.9 | 73.1 | 0.07 |
| 65-74 | 12.9 | 10.8 | 9.0 | 14.5 | 14.0 | 12.0 | 20.8 | 16.4 |  |
| 75-84 | 10.4 | 10.7 | 6.4 | 5.5 | NR^b^ | 4.0 | 9.4 | 10.4 |  |
| 85+ | 0.8 | 1.2 | NR^b^ | NR^b^ | NR^b^ | NR^b^ | NR^b^ | NR^b^ |  |
| Insurance |  |  |  |  |  |  |  |  |  |
| Medicare | 29.1 | 26.8 | 39.6 | 46.3 | 21.6 | 23.6 | 36.7 | 37.5 | <0.01 |
| Medicaid | 35.5 | 44.2 | 29.2 | 32.4 | 47.3 | 54.2 | 20.4 | 31.2 |  |
| Private insurance | 28.3 | 21.3 | 29.2 | 18.5 | 23.0 | 22.2 | 38.8 | 25.0 |  |
| Self-pay | 7.0 | 7.7 | NR^b^ | 2.8 | 8.1 | NR^b^ | NR^b^ | 6.3 |  |
| Income quartile |  |  |  |  |  |  |  |  |  |
| I | 29.5 | 33.2 | 51.3 | 45.4 | 48.6 | 40.3 | 18.7 | 27.7 | <0.01 |
| II | 24.1 | 27.3 | 18.6 | 27.8 | 12.9 | 26.4 | 25.0 | 21.5 |  |
| III | 23.1 | 22.8 | 16.8 | 16.7 | 25.7 | 23.6 | 20.8 | 24.6 |  |
| IV | 23.3 | 16.6 | 13.3 | 10.2 | 12.9 | 9.7 | 35.4 | 26.2 |  |
| **Hospital characteristics** |  |  |  |  |  |  |  |  |  |
| Location/teaching status |  |  |  |  |  |  |  |  |  |
| Rural | 2.3 | 1.7 | NR^b^ | NR^b^ | NR^b^ | NR^b^ | NR^b^ | NR^b^ | 0.22 |
| Urban nonteaching | 5.8 | 7.1 | 2.6 | 10.0 | NR^b^ | NR^b^ | NR^b^ | 6.0 |  |
| Urban teaching | 91.9 | 91.2 | 96.5 | 88.2 | 98.7 | 100 | 96.2 | 94.0 |  |
| Bed size |  |  |  |  |  |  |  |  |  |
| Small | 7.9 | 8.4 | NR^b^ | 6.4 | 6.4 | NR^b^ | 5.7 | 4.5 | 0.02 |
| Medium | 19.9 | 21.6 | 15.8 | 13.6 | 12.8 | 21.3 | 18.9 | 10.4 |  |
| Large | 72.1 | 70.0 | 83.3 | 80.0 | 80.8 | 76.0 | 75.5 | 85.1 |  |
| Region |  |  |  |  |  |  |  |  |  |
| Northeast | 18.8 | 16.0 | 17.5 | 12.7 | 21.8 | 24.0 | 32.1 | 29.9 | <0.01 |
| Midwest | 26.6 | 28.3 | 30.7 | 22.7 | 9.0 | 8.0 | 11.3 | 9.0 |  |
| South | 37.1 | 42.3 | 44.7 | 50.0 | 29.5 | 30.7 | 22.6 | 22.4 |  |
| West | 17.4 | 13.3 | 7.0 | 14.5 | 39.7 | 37.3 | 34.0 | 38.8 |  |
| Elective admission | 43.2 | 41.2 | 28.1 | 35.5 | 35.1 | 50.7 | 43.4 | 50.7 | 0.01 |
| Weekend admission | 15.1 | 14.8 | 24.6 | 14.5 | 16.7 | 13.3 | 17.0 | 11.9 | 0.26 |
| **Clinical characteristics** |  |  |  |  |  |  |  |  |  |
| Elixhauser comorbidity index | 6 (5-8) | 6 (5-8) | 7 (6-8) | 7 (5-8) | 6 (4-7) | 7 (5-8) | 6 (5-8) | 6 (5-8) | <0.01 |
| Charlson comorbidity index | 2 (1-3) | 1 (1-3) | 3 (1-5) | 2 (1-4) | 1 (0-3) | 2 (1-4) | 2 (1-3) | 2 (1-3) | <0.01 |
| 0 | 29.4 | 31.1 | 16.7 | 14.5 | 34.6 | 24.0 | 22.6 | 26.9 | <0.01 |
| 1 | 25.7 | 32.3 | 21.9 | 26.4 | 23.1 | 34.7 | 30.2 | 31.3 |  |
| 2 | 15.7 | 17.0 | 11.4 | 15.5 | 17.9 | 16.0 | 13.2 | 16.4 |  |
| ≥3 | 29.1 | 19.5 | 50.0 | 43.6 | 24.4 | 25.3 | 34.0 | 25.4 |  |
| **Individual comorbidities** |  |  |  |  |  |  |  |  |  |
| Diabetes mellitus | 13.9 | 8.4 | 29.8 | 31.8 | 19.2 | 18.7 | 17.0 | 14.9 | <0.01 |
| Hypertension | 46.3 | 37.3 | 65.8 | 67.3 | 50.0 | 44.0 | 64.2 | 40.3 | <0.01 |
| Dyslipidemia | 22.7 | 15.6 | 28.1 | 29.1 | 21.8 | 21.3 | 35.8 | 19.4 | <0.01 |
| Nicotine/tobacco use | 46.6 | 52.1 | 41.2 | 31.8 | 44.9 | 42.7 | 35.8 | 25.4 | <0.01 |
| Alcohol abuse | 6.4 | 2.7 | 4.4 | NR^b^ | NR^b^ | NR^b^ | NR^b^ | NR^b^ | <0.01 |
| Drug abuse | 36.6 | 51.1 | 21.9 | 22.7 | 34.6 | 32.0 | 20.8 | 28.4 | <0.01 |
| Obesity | 10.1 | 10.9 | 13.2 | 24.5 | 9.0 | 12.0 | 9.4 | 9.0 | 0.01 |
| Coronary artery disease | 21.7 | 11.7 | 28.9 | 20.9 | 17.9 | 20.0 | 30.2 | 10.4 | <0.01 |
| Peripheral vascular disease | 9.2 | 5.7 | 21.9 | 14.5 | 3.8 | 5.3 | NR^b^ | 11.9 | <0.01 |
| Atrial fibrillation/flutter | 39.7 | 29.3 | 36.0 | 38.2 | 33.3 | 34.7 | 50.9 | 41.8 | <0.01 |
| Congestive heart failure | 49.7 | 43.1 | 66.7 | 62.7 | 52.6 | 50.7 | 58.5 | 49.3 | <0.01 |
| Renal failure | 23.1 | 13.7 | 42.1 | 30.9 | 21.8 | 18.7 | 24.5 | 19.3 | <0.01 |
| Dialysis dependent | 1.7 | 0.7 | 6.1 | 7.3 | 7.7 | NR^b^ | NR^b^ | NR^b^ | <0.01 |
| Liver disease | 18.7 | 20.8 | 14.9 | 13.6 | 17.9 | 17.3 | 22.6 | 20.9 | 0.54 |
| Chronic pulmonary disease | 16.7 | 16.9 | 23.7 | 20.0 | 10.3 | 16.0 | 15.1 | 13.4 | 0.36 |
| Obstructive sleep apnea | 11.4 | 5.4 | 14.9 | 11.8 | 5.1 | 10.7 | NR^b^ | 4.5 | <0.01 |
| Coagulopathy | 43.8 | 42.4 | 53.5 | 37.3 | 42.3 | 52.0 | 47.2 | 41.8 | 0.21 |
| Cancer | 1.6 | 1.0 | NR^b^ | NR^b^ | NR^b^ | NR^b^ | NR^b^ | NR^b^ | 0.72 |
| Malnutrition | 15.3 | 13.7 | 12.3 | 10.9 | 11.5 | 16.0 | NR^b^ | 7.5 | 0.21 |
| Dementia | NR^b^ | 0.3 | NR^b^ | NR^b^ | NR^b^ | NR^b^ | NR^b^ | NR^b^ | 0.56 |
| Depression | 12.0 | 22.3 | 12.3 | 17.3 | 14.1 | 10.7 | 5.7 | 11.9 | <0.01 |
| **Previous history** |  |  |  |  |  |  |  |  |  |
| Myocardial infarction | 3.6 | 1.6 | 7.0 | 3.6 | NR^b^ | 4.0 | NR^b^ | NR^b^ | 0.02 |
| Stroke/TIA | 4.0 | 4.1 | 7.0 | 3.6 | 3.8 | 5.3 | NR^b^ | 6.0 | 0.59 |
| Cardiac arrest | 0.8 | 0.5 | NR^b^ | NR^b^ | NR^b^ | NR^b^ | NR^b^ | NR^b^ | 0.51 |
| PCI | 4.4 | 1.3 | 4.4 | NR^b^ | NR^b^ | NR^b^ | NR^b^ | NR^b^ | <0.01 |
| CABG | 3.4 | 2.4 | 2.6 | NR^b^ | 3.8 | NR^b^ | NR^b^ | NR^b^ | 0.37 |
| ICD | 4.7 | 1.3 | 10.5 | 8.2 | NR^b^ | NR^b^ | NR^b^ | 4.5 | <0.01 |
| PPM | 4.7 | 7.5 | NR^b^ | NR^b^ | 6.4 | 9.3 | NR^b^ | 10.4 | 0.01 |

*Note*. Data presented as median (IQR) or %.

^a^Asian or Pacific Islander, Native American, and Other.

^b^Cell counts <11 are not reportable (NR) per HCUP guidelines.

*Abbreviations.* TIA=transient ischemic attack; PCI=percutaneous coronary intervention; CABG=coronary artery bypass grafting; ICD=implantable cardioverter-defibrillator; PPM=permanent pacemaker; IQR=interquartile range.
