# Supplementary material for: Racial, ethnic, and sex disparities in the utilization and outcomes of tricuspid valve surgery
Source: Ann Med Surg (Lond). 2024 Jun 19;86(8):4368–76. doi: 10.1097/MS9.0000000000002203 (PMC11305732; doi:10.1097/MS9.0000000000002203)
Supplement: Supplementary file 5 [file ms9-86-4368-s005.docx]

**Table S4.** Adjusted in-hospital outcomes following isolated tricuspid valve surgery stratified by race/ethnicity and sex

|  | White | | Black | | Hispanic | | Other^a^ | |
| --- | --- | --- | --- | --- | --- | --- | --- | --- |
|  | Men  (*n*=3,210) | Women  (*n*=4,870) | Men  (*n*=570) | Women  (*n*=550) | Men  (*n*=390) | Women  (*n*=375) | Men  (*n*=265) | Women  (*n*=335) |
| **Death** | Ref. | 0.70 (0.39-1.26) | 0.69 (0.26-1.87) | 1.01 (0.40-2.53) | 0.93 (0.27-3.11) | 0.67 (0.18-2.52) | 0.51 (0.10-2.48) | 1.43 (0.51-3.98) |
| **Complications** |  |  |  |  |  |  |  |  |
| Heart block | Ref. | 1.03 (0.81-1.33) | 0.84 (0.51-1.38) | 0.79 (0.47-1.31) | 0.98 (0.54-1.77) | 1.02 (0.59-1.78) | 0.96 (0.47-1.96) | 1.14 (0.62-2.08) |
| Permanent pacemaker | Ref. | 1.30 (0.96-1.77) | 0.74 (0.36-1.49) | 0.82 (0.43-1.57) | 1.48 (0.78-2.82) | 1.05 (0.51-2.12) | 1.11 (0.45-2.72) | 1.51 (0.72-3.13) |
| Stroke | Ref. | 0.74 (0.33-1.65) | 1.39 (0.42-4.51) | 0.53 (0.16-1.71) | 1.05 (0.12-8.99) | 1.40 (0.35-5.62) | 0.29 (0.05-1.95) | 2.44 (0.98-4.93) |
| Acute kidney injury | Ref. | **0.65 (0.50-0.83)** | 1.14 (0.63-2.07) | **0.48 (0.27-0.87)** | 0.92 (0.52-1.61) | **0.32 (0.18-0.59)** | 0.84 (0.40-1.77) | **0.54 (0.27-0.92)** |
| Major bleeding | Ref. | 0.90 (0.71-1.13) | 1.29 (0.79-2.12) | 0.79 (0.50-1.26) | 1.20 (0.67-2.13) | 0.74 (0.42-1.07) | 1.05 (0.53-2.05) | 1.34 (0.78-2.31) |
| Blood transfusion | Ref. | 1.16 (0.95-1.43) | **1.91 (1.09-3.34)** | 1.33 (0.77-2.30) | 1.48 (0.79-2.76) | 1.21 (0.62-2.36) | 1.70 (0.79-3.66) | 1.52 (0.78-2.97) |
| Vascular complications | Ref. | 0.91 (0.58-1.44) | 1.34 (0.61-2.96) | 1.12 (0.43-2.87) | 0.40 (0.09-1.92) | 1.48 (0.58-3.78) | 0.25 (0.03-2.22) | 0.47 (0.11-2.03) |

*Note*. Data presented as aOR (95% CI) or %. The bold values indicate statistical significance. Outcomes adjusted for age, insurance, income, hospital location and teaching status, bed size, region, type of admission, Elixhauser and Charlson index scores, and relevant comorbidities.

^a^Asian or Pacific Islander, Native American, and Other.

*Abbreviations*. aOR=adjusted odds ratio; CI=confidence interval.
